# Analysis of knee flexion characteristics and how they alter with the onset of knee osteoarthritis: a case control study

**DOI:** 10.1186/1471-2474-14-169

**Published:** 2013-05-21

**Authors:** Ian McCarthy, Diana Hodgins, Amit Mor, Avi Elbaz, Ganit Segal

**Affiliations:** 1 European Technology for Business Ltd, Codicote, UK; 2 Royal National Orthopaedic Hospital, Stanmore, UK; 3 AposTherapy Research Group, 1st Abba Even Blvd, Herzliya 46733, Israel

**Keywords:** Osteoarthritis, Gait, Electronic measurement systems

## Abstract

**Background:**

The purpose of this study was to examine the differences in gait profile between patients with knee osteoarthritis (OA) and healthy control and to create motion characteristics that will differentiate between them.

**Methods:**

Twenty three patients diagnosed with knee OA and 21 healthy matched controls underwent a gait test using a sensor system (gaitWALK). Gait parameters evaluated were: stride duration, knee flexion range of motion (ROM) in swing and stance. *T*-Test was used to evaluate significant differences between groups (P < 0.05).

**Results:**

Patients with knee OA had significant lower knee flexion ROM (10.3° ± 4.0°) during stance than matched controls (18.0° ± 4.0°) (p < 0.001). Patients with knee OA had significant lower knee flexion ROM (54.8° ± 5.5°) during swing than matched controls (61.2° ± 6.1) (p = 0.003). Patients with knee OA also had longer stride duration (1.12 s ± 0.09 s) than matched controls (1.06 s ± 0.11 s), but this was not statistically significant (p = 0.073). Motion characteristics differentiate between a patient with knee OA and a healthy one with a sensitivity of 0.952 and a specificity of 0.783.

**Conclusions:**

Significant differences were found in the gait profile of patients with knee OA compared to matched control and motion characteristics were identified. This test might help clinicians identify and evaluate a knee problem in a simple gait test.

## Background

Osteoarthritis (OA) is by far the most common form of arthritis. Around 2.5% of the adult population suffer from OA of the hip or knee, most of whom are over 45 and this increases to 10% for women over 75 [1]. The main symptoms are pain and limitation in function, which normally leads to changes in gait patterns to accommodate the pain [2].

Because the prevalence of knee OA is high and increasing in the adult population, a means of early diagnosis is being sought. Current diagnosis in an orthopaedic clinic is done using a standard X-ray machine, and the level of degeneration is assessed. One common grading score for hip and knee OA is the Kellgren and Lawrence score [3]. However, it is very difficult to quantify this, particularly at the early stages. Previous studies have reported that changes to the knee joint occur even before radiographic changes are detected [4-6]. Shakoor et al. reported in 2003 that knee loading at the contralateral limb increases following hip arthroplasty and increase the rate of developing knee OA [6]. Furthermore, previous studies have stressed the poor correlation between radiographic changes and symptoms of pain and function [7,8]. Researchers and clinicians are seeking a method of ascertaining the functional severity of the OA, which ideally can be used alongside X-ray data, to detect early stage OA [9].

Biomechanics plays an important role in the progression of knee OA and many studies have been carried out in gait laboratories to ascertain which parameters are affected for people suffering with knee OA compared to healthy subjects [2,10]. Many papers have concluded that reduction in gait velocity is a prominent change in patients with knee OA compared to healthy controls [11,12]. However, it is also recognised that there are many other conditions where gait velocity is reduced, including age, and therefore velocity itself cannot be a good discriminator for OA [13]. There is comprehensive evidence that the maximum knee flexion angle in stance is reduced (the limb is more extended) with the severity of the OA when compared to normal subjects, and some suggest that this is in order to care for and prevent knee OA progress [14-16].

Most studies of knee joint kinematics and kinetics have been performed in a dedicated gait laboratory. Although providing detailed information, the procedure is expensive and time-consuming, requiring subjects to be brought to the laboratory [17]. There is a lack of measuring systems that may be used in clinics or taken out into the community. Portable walkways provide information of the spatio-temporal characteristics of gait [18,19]. However, this does not measure knee joint movement, which may supply additional information concerning the patient’s condition. Inertial measurement units (IMUs) are now being used more commonly to assess gait, and do enable knee kinematics to be investigated in an out-patient clinic [20].

The growing interest in early detection of knee OA (prior to structural changes) and objective functional assessment of patients with knee OA were the basis for this study. The current study had three aims. First, to provide further evidence to support the claim that patients with knee OA demonstrate altered gait pattern compared to matched controls, and specifically to examine the differences between groups in knee flexion angle during the stance and the swing phases. The second aim was to demonstrate that knee motion can be measured using a sensor based system that can be used in a clinic, rather than in a gait laboratory. Finally, the third aim was to draw a set of criteria that would differentiate between patients with knee OA and matched controls, based on the knee flexion angle during the stance phase.

## Methods

Overall, 44 people participated in this study. Twenty three patients with a mean ± sd age of 65.1 ± 7.7 years and a mean BMI of 28.7 ± 3.7 were diagnosed with medial compartment knee OA (14 females, 9 males). 15 patients had bilateral knee OA and 9 patients had unilateral knee OA. Average duration of symptoms was 12.3 ± 6.5 months (6–24 months), which is thought to represent a cohort of patients with early signs of knee OA. Inclusion criteria were: 1. Patients suffering from symptomatic knee OA at the medial compartment for at least six months, fulfilling the American College of Rheumatology (ACR) clinical criteria for OA of the knee [21]. According to Altman et al., clinical classification of a patient with knee OA will be if the patient present at least 3 of the following 6 clinical findings: age >50 years, morning stiffness <30 minutes duration, crepitus on active motion, tenderness of the bony margins of the joint, bony enlargement noted on examination, and a lack of palpable warmth of the synovium.

Exclusion criteria were: 1. Acute septic arthritis; 2. Inflammatory arthritis; 3. Corticosteroid injection within 3 months of the study; 4. Avascular necrosis of the knee; 5. History of knee buckling or recent knee injury; 6. Joint replacement; 7. Neuropathic arthropathy; 8. History of pathological osteoporotic fracture; 11. Symptomatic degenerative arthritis in lower limbs joints other than the knees. The head researcher used these criteria to determine the inclusion or exclusion of patients from the existing database. Patients were recruited from the AposTherapy Center, Herzliya, Israel. The study protocol was approved by the institutional Helsinki Committe of Assaf Harofeh Medical Center, Zerifin, Israel. Twenty one healthy people served as controls. Their mean ± sd age was 71.3 ± 6.1 years (17 females, 4 males), and mean ± sd BMI was 25.5 ± 2.9. These people were recruited from staff and volunteers working at the Royal National Orthopaedic Hospital, London, UK. The study protocol was approved by the University College London research ethics committee. Consent was obtained from both the patients and healthy volunteers.

### Measuring system

**Figure 1:**
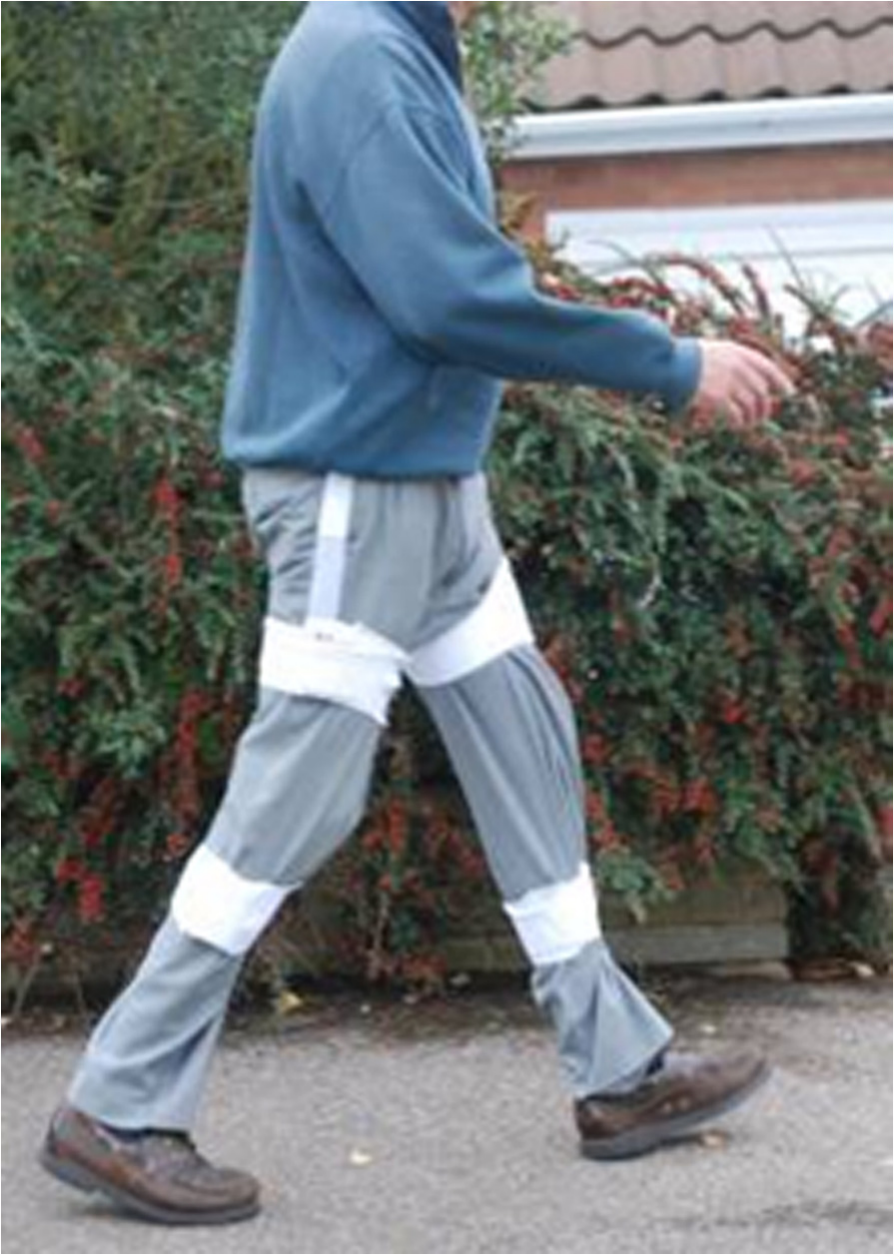
Volunteer walking with the IMUs strapped to the thigh and shank.

The gait profile was measured using inertial sensors mounted onto the lower limbs and associated analysis software, provided by European Technology for Business Ltd [22]. The system used in this trial comprised four sensor modules, a laptop and four straps. The sensors are IMUs and contain 3 orthogonal gyroscopes and 3 orthogonal accelerometers, as used by Cooper et al. in an earlier study on joint angles [23]. The sensors contain a precision clock and a memory storage device card (SD card) and data is gathered from each sensor at 102.4Hz. Figure 1 shows a volunteer walking with the 4 sensors inserted into pockets integrated into the straps.

### Protocol

The four sensors were time stamped and synchronised using the Poseidon software on the laptop. The sensors were then disconnected from the laptop. Each person was asked to stand whilst the straps were put on to the thigh and calf of each leg. The location of the straps on the calf was at the level of the belly of the gastrocnemius muscle, with the sensor being located on the lateral side of the calf. Location of the straps on the thigh was on the proximal end of the thigh, just bellow the greater trochanter, with the sensors being located on the lateral side of the thigh. Then one sensor was switched on and located into the appropriate pocket. The strap was then fastened securely over the pocket. This was repeated for all four sensors. The sensors were all aligned to the line of the limb segment by eye, on both the thigh and calf. No attempt was made to align them exactly as only the range of motion (ROM) of the knee flexion angle in swing and stance over a stride were required for this study. Any misalignment of the sensor to the segment would introduce an offset to the joint angle, but would not affect the ROM or profile. Any misalignment around the body (from the sagittal towards the coronal plane) would introduce a small error in the ROM (<2% for ±10^0^). The person was then asked to stand stationary for 5 seconds, to calibrate the sensors. The patient was asked to walk at his/herself-selected speed on a 20 meters level surface. At the end of the test the sensors were removed from their pockets, switched off and then connected to the laptop for analysis.

### Data analysis

**Figure 2:**
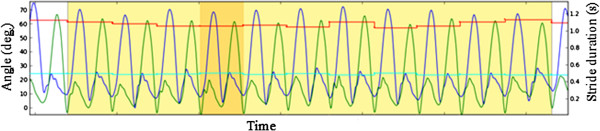
**Typical knee angle profile obtained during the test procedure. **The darker shaded area shows the most typical stride during the test, which is used for subsequent analysis.

The analysis of the data was done using the Poseidon software installed on the laptop [22]. The software calculates the knee joint angle for the entire test. From this the section was chosen for analysis, where the person is walking steadily for at least 7 strides, which is approximately 8 m. The software then calculates the typical stride, i.e. the stride with lowest error to all other strides, shaded darker in the plot in Figure 2. Previous studies confirmed that stride-to-stride variability during walking is low [24] and hence the analysis of the typical stride is considered valid. The following parameters were calculated: Knee flexion ROM during stance phase (deg), knee flexion ROM (deg) during swing phase, and stride time (s).

### Statistical analysis

Statistical analysis was performed using SPSS Version 19. Group differences for age, BMI and stride duration were assessed using Student’s *t*-test. Differences for knee angle characteristics were assessed using ANOVA to compare controls and OA and non-OA knees in the patients. Receiver operator characteristic (ROC) analysis was performed on data from controls and OA knees to determine the extent to which measurements could differentiate between the two groups.

## Results

There was no significant difference in gender distribution between patients with knee OA and healthy controls (λ^2^ = 2.12, p >0.1). A significant difference between patients with knee OA and healthy controls was found in BMI. Patients with knee OA had higher a BMI value compared to healthy controls (28.7 ± 3.74 and 25.5 ± 2.9, respectively, p = 0.004). There was no significant difference in BMI between females and males with knee OA as well as between healthy females and healthy males. In addition, there was no significant difference in age between healthy females and healthy males. There was, however a significant difference in age between females and males with knee OA. Males with knee OA were slightly younger than females with knee OA (60.0 ± 5.0 and 68.4 ± 7.4, respectively).

**Figure 3:**
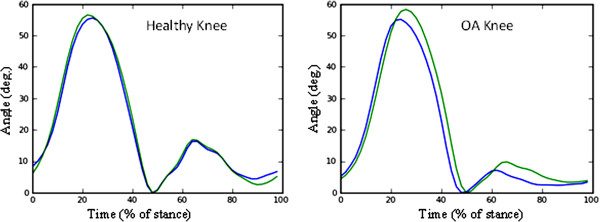
**Examples of a typical stride for a control subject and one with bilateral osteoarthritis.** The blue trace is the left knee and the green trace is the right knee.

The knee profile was examined for the 21 healthy volunteers and 23 patients. An example is provided for one of each in Figure 3.

Data are summarised in Table 1. High correlation was found between left and right knees for both controls and patients with bilateral OA (r ~ 0.82), hence left and right data were averaged for these subjects. For patients with unilateral OA, data for the OA limb was included with the data for subjects with bilateral OA, and the data for the unaffected limb was analysed separately. Overall, 39 OA knees, 42 control knees and 9 non-OA knees were analysed. ANOVA showed statistically significant differences between the groups (controls, OA knees and non-OA knees in OA patients) for both swing and stance flexion ROM; post-hoc analysis confirmed differences between controls and OA knees (p < 0.001 for stance phase and p = 0.003 for swing phase), but no statistical difference between the non-OA knees and either the controls or OA knees. There was no statistically significant difference in stride duration between the control and OA subjects (p = 0.073), although patients with knee OA walked slower.

**Table 1:** Gait profile differences between patients with knee OA and healthy controls

	**Duration (s)**	**Flexion ROM in swing (°)**	**Flexion ROM in stance (°)**
OA knees	1.12 (0.09)	54.8 (5.5)*	10.25 (4.0)*
Non OA knees	-	57.6 (4.6)	14.08 (4.3)
Controls	1.06 (0.11)	61.2 (6.1)*	18.04 (4.0)*

* Indicates statistically significant difference between OA and control knees (p < 0.001).

Results are presented as mean (sd).

Gender analysis was carried in order to examine whether the aforementioned significant differences in knee angles are related to gender. Similar trends were found in both females and males with differences in knee flexion ROM during swing (P = 0.006 for females, P = 0.08 for males) and knee flexion ROM during stance (P = 0.001 for females, P = 0.02 for males). This suggests that gender was not a confounder to the results of this study.

**Figure 4:**
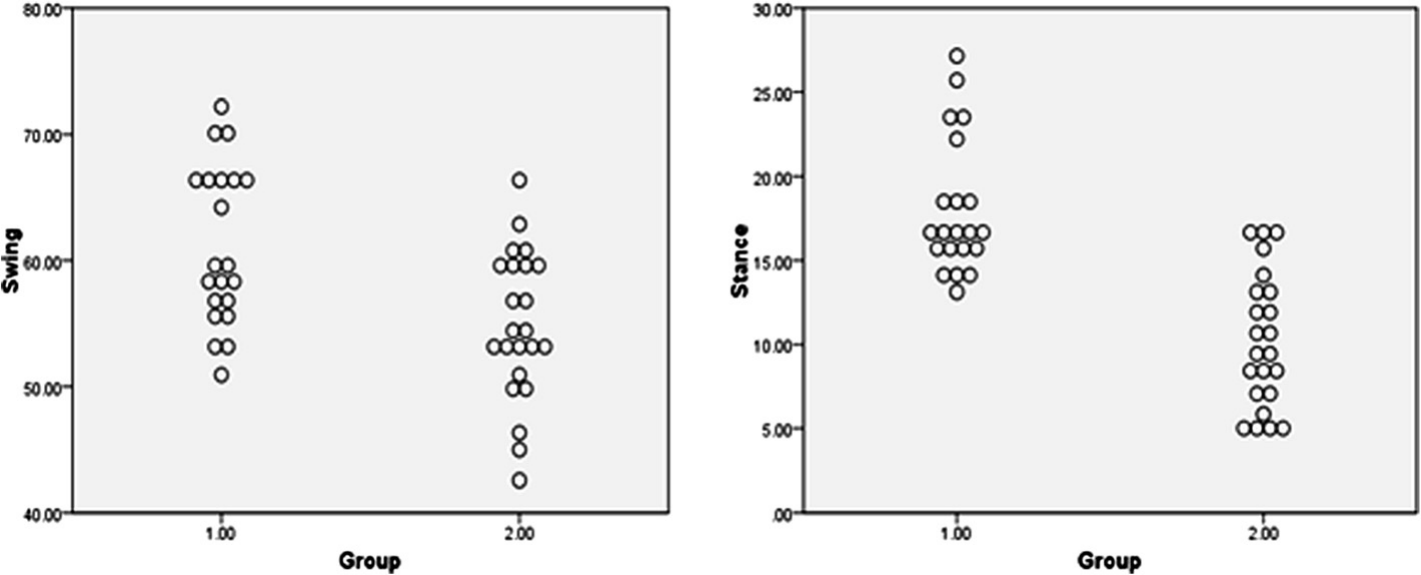
Scatter plot of ROM knee flexion in swing (°) and ROM knee flexion in stance (°) for typical stride: controls = Group 1; OA = Group2.

Scatter plots of the knee flexion ROM data for the OA and control knees are shown in Figure 4; it can be seen that there is considerable overlap between the controls (group 1) and those with knee OA (group 2) for the knee flexion ROM in swing, whereas there is little overlap between the two groups for knee flexion ROM in stance.

**Figure 5:**
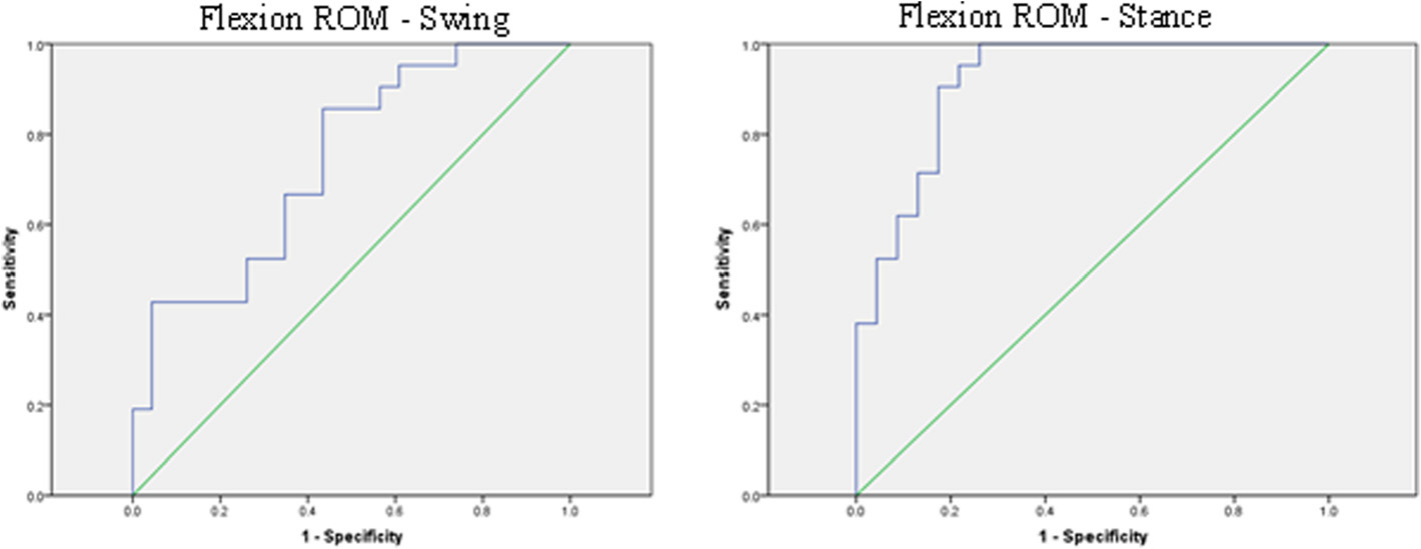
**ROC plots for knee flexion ROM in swing (left plot) and stance (right plot). **The areas under the curves are 0.741 (swing) and 0.913 (stance).

ROC analysis of the data is shown in Figure 5, with the area under the curve being 0.914 for stance knee flexion ROM, and 0.741 for swing knee flexion ROM. The ROC analysis indicated that a cut-off value of 13.6° of flexion ROM in stance could discriminate between controls and patients with OA with a specificity of 0.952 and a sensitivity of 0.783.

ROC analysis was also preformed on females and males separately. For females, the area under the curve being 0.987 for stance knee flexion ROM, and 0.773 for swing knee flexion ROM. For males, the area under the curve being 0.889 for stance knee flexion ROM, and 0.722 for swing knee flexion ROM. This indicates that stance knee flexion ROM is a strong predictor of OA even when male and female data are analysed separately. We also examined the relationship between stance knee flexion ROM and BMI. Within each group, there was a very slight but non-significant decrease in knee stance flexion ROM with BMI (ROM = -0.277*BMI + 16.76 (OA); ROM = -0.248*BMI + 24.34 (control)). Although neither of these regressions are significant, the similarity of the regression coefficients and the difference in the intercepts suggests that difference in BMI is not a confounding factor.

## Discussion

The purpose of the current study was to characterize the gait profile of patients with knee OA compared to matched controls using a new measuring system that can be applied in clinic settings rather than laboratory settings. In addition, this study tried to determine a set of motion characteristics that would differentiate between a healthy person and a person suffering from knee OA. The results showed significant differences between the gait profile of patients with knee OA compared to matched controls, further supporting existing literature [2,10,14-16,25]. Furthermore, a specific gait parameter was found to be a good discriminator between a healthy knee motion and a pathologic one.

Patients with knee OA showed decreased stride time compared to matched control, though this did not reach statistical significance (p = 0.073). This finding supports previous papers that have reported that patients with knee OA walk slower than matched controls [16,26]. Patients with knee OA showed decreased knee flexion ROM during stance phase (39.7%) compared to controls. This gait change has been reported in previous studies that examined the changes in gait patterns between patients with knee OA and healthy controls [14-16]. This difference is sought to be a compensation strategy of knee OA patients in response to pain; the peak flexion in stance coincides with the peak knee flexion moment, which has also been shown to be reduced in OA [15]. This may be achieved by reduction of quadriceps force, known as quadriceps avoidance strategy, and is consistent with observations of quadriceps weakness in patients with OA [27]. There is disagreement in the current literature regarding the correlation between gait velocity and knee motion. Previous studies have reported that patients with knee OA demonstrate reduced knee motion during walking compared to healthy controls alongside the reduction in gait velocity [11]. A more recent study, however by Bejek et al. 2006, examined the effect of different walking speed on gait parameters in healthy subjects and in patients with knee OA [12]. Their results supported previous studies and showed that patients with knee OA demonstrate reduced knee motion compared to healthy controls in varying gait speeds. Interestingly, knee ROM and maximum knee flexion during walking changed significantly with changes in gait velocity within healthy patients, but not within knee OA patients. Based on their results an OA knee will present reduced motion during walking regardless to gait velocity compared to a healthy knee, hence we assumed knee motion is a better indicator to detect a pathological knee. The results of this study supported this assumption.

Few studies have investigated the differences in knee angle during the swing phase between patients with knee OA and healthy controls. In the current study, a statistically significant difference between group means for the knee joint ROM during swing phase was found. A considerable overlap between the groups in the above mentioned parameter was seen (Figure 4), leading to the conclusion that this measurement is poor at discriminating between knee OA patients and controls. Kaufman et al. reported a non-significant difference in swing flexion ROM angle [2], though the magnitude of the difference appears similar to that reported in this paper, where significant difference has been shown. It has been suggested that reduced knee flexion ROM in the swing phase (characterised as ‘stiff knee’ gait pattern) is caused by over activity of the rectus femoris [28]. Future studies should further examine the differences between patients with knee OA and healthy controls in knee joint angle during the swing phase of walking.

There is growing evidence that knee OA begins before radiographic changes are detected [4-6]. From a biomechanical point of view this includes changes in knee loading [6] and muscle activation [29]. Researchers therefore are trying to find tests that will help in the early detection of knee OA in order to start treatment as early as possible and delay, stop or even reverse disease progression. Three dimensional gait analysis has been shown to detect differences between knee OA patients and controls and is considered a reliable test [2,10,14-16,25]. This analysis, however is cumbersome both from the patients point of view (long test, usually with minimal wearing) and from the examiner point of view (long test, numerous data, difficulty in understanding the report etc.) [17]. Recently, Elbaz et al. have suggested single limb support (a spatio-temporal parameter) to be an indicator for disease severity [9]. Spatio-temporal parameters however, do not include measurements of joint motion and might miss important information. The current study suggest a new test that measures knee motion in a simple way that can be applied widely in clinical settings. The current study found one parameter, knee flexion ROM during stance phase, which can be a discriminator between patients with knee OA and matched healthy controls. A threshold value has been determined and was able to detect 78.3% of OA knees and 95.2% of healthy knees. Future studies should further investigate and support this finding.

Some limitation should be acknowledged. First, a radiographic evaluation of knee joint structural changes was missing. Patients were diagnosed with knee OA based on the American College of Rheumatology (ACR) clinical criteria [24]. We recommend that future studies will examine the differences in gait profile with this system in different knee joint deformity groups, as determined by x-ray. Secondly, this study showed that knee flexion ROM can be a good discriminator between patients with knee OA and healthy controls, even in cases were gait velocity is similar. This study did not discuss the gait profile of other pathological population. Theoretically, two different pathologic populations might present the same altered knee motion compared to healthy people. This test, therefore should always accompany a clinical assessment and anamnesis alongside other evaluation tests (X-ray, objective spatio-temporal gait analysis). Furthermore, future studies should examine and characterized the knee profile of other pathologies and compare them with the knee profile of an OA knee. It is also acknowledged that measurements were performed at two different sites, with the OA patients measured in Israel, and the age-matched controls in the UK. We have, however, compared data on comparable young age-matched controls measured at the two sites, and found no differences in any of the gait parameters, and therefore do not consider that the use of two different sites has introduced any systematic error.

## Conclusions

This analysis of people with knee OA has confirmed previous findings that knee flexion ROM on load is reduced with the onset of knee OA. It has also demonstrated that this parameter can serve as a good discriminator between patients with knee OA and controls. It might also be sensitive enough to detect changes over time in response to treatment. Finally, this test can be applied in clinical environment.

## Abbreviations

OA: Osteoarthritis; IMUs: Inertial measurement units; SD card: Storage device card; ROM: Range of motion; ROC: Receiver operator characteristic.

## Competing interests

Diana Hodgins is the developer and owner of the gaitWALK system.

## Authors’ contribution

DH conception and design, drafting the article, final approval. IM data collection, drafting the article, statistical analysis, final approval. AM revising the article, final approval. AE revising the article, final approval. GS data collection, drafting the article, final approval. All authors read and approved the final manuscript.

## Pre-publication history

The pre-publication history for this paper can be accessed here:

http://www.biomedcentral.com/1471-2474/14/169/prepub
